# The laboratory health system and its response to the Ebola virus disease outbreak in Liberia

**DOI:** 10.4102/ajlm.v5i3.509

**Published:** 2016-10-31

**Authors:** Stephen B. Kennedy, Christine L. Wasunna, John B. Dogba, Philip Sahr, Candace B. Eastman, Fatorma K. Bolay, Gloria T. Mason, Mark W.S. Kieh

**Affiliations:** 1 Incident Management System, Emergency Operations Center, Ministry of Health, Monrovia, Liberia; 2 Partnership for Research on Ebola Virus in Liberia, Liberia-US Clinical Research Partnership Program, First Floor, John F. Kennedy Medical Center, Monrovia, Liberia; 3 National Reference Laboratory, Ministry of Health, Charlesville, Margibi County, Liberia; 4 Africabio Enterprises, Inc., Payne Avenue, Sinkor, Monrovia, Liberia; 5 Liberia Institute for Biomedical Research, Ministry of Health, Charlesville, Margibi County, Liberia; 6 National Research Ethics Board, Partnership for Research on Ebola Virus in Liberia, First Floor, John F. Kennedy Medical Center, Monrovia, Liberia

## Abstract

The laboratory system in Liberia has generally been fragmented and uncoordinated. Accordingly, the country’s Ministry of Health established the National Reference Laboratory to strengthen and sustain laboratory services. However, diagnostic testing services were often limited to clinical tests performed in health facilities, with the functionality of the National Reference Laboratory restricted to performing testing services for a limited number of epidemic-prone diseases. The lack of testing capacity in-country for Lassa fever and other haemorrhagic fevers affected the response of the country’s health system during the onset of the Ebola virus disease (EVD) outbreak. Based on the experiences of the EVD outbreak, efforts were initiated to strengthen the laboratory system and infrastructure, enhance human resource capacity, and invest in diagnostic services and public health surveillance to inform admittance, treatment, and discharge decisions. In this article, we briefly describe the pre-EVD laboratory capability in Liberia, and extensively explore the post-EVD strengthening initiatives to enhance capacity, mobilise resources and coordinate disaster response with international partners to rebuild the laboratory infrastructure in the country. Now that the EVD outbreak has ended, additional initiatives are needed to revise the laboratory strategic and operational plan for post-EVD relevance, promote continual human resource capacity, institute accreditation and validation programmes, and coordinate the investment strategy to strengthen and sustain the preparedness of the laboratory sector to mitigate future emerging and re-emerging infectious diseases.

## Introduction

The first laboratory policy in Liberia was completed in 2010 through the efforts of the National Diagnostic Unit–Laboratory Technical Working Group. This led to the development of a comprehensive three-year Laboratory Strategic and Operational Plan (2011–2013) for the effective operation, management and sustainability of the laboratory system in Liberia.^1^ However, this plan was not fully instituted due to the lack of adequate funding for its implementation and operationalisation as a key pillar for the rebuilding of the resilient health delivery system of the country. The lack of adequate funding and limited human resources to support diagnostic and laboratory services remain as major challenges.

With the desired goal of disease surveillance and epidemic control of the Ministry of Health, the major emphasis of the Laboratory Strategic and Operational Plan was to develop a sustainable plan to build and strengthen the national laboratory system and its governance structure. The need for alignment of resources and coordination to develop an integrated laboratory system with clear definition of roles and responsibilities became a priority of the Ministry of Health. Initiatives were set to review the current situation to avoid duplication, reduce the turnaround time of test results and strengthen an effective and efficient reporting mechanism. Importantly, the critical need for laboratory human resource development to adequately respond to future outbreaks was highlighted.

## Pre-Ebola virus disease laboratory system

Prior to the Ebola virus disease (EVD) outbreak in Liberia, the structure of the system regulating the laboratory environment was fragmented and not well coordinated. Laboratory subunits within the Ministry of Health, including the National Reference Laboratory, the National Diagnostics Unit and the National Blood Safety Program, were poorly coordinated. This led to duplication and overlapping of functions as there was a poorly-defined demarcation of duties and responsibilities among these subunits. A variety of equipment platforms were frequently out of service, with limited capacity for repairs and preventive maintenance. Moreover, the stock-out of needed reagents and consumables often led to an interruption of diagnostic services, such as CD4 testing, clinical chemistry and haematology, throughout the country.

Diagnostic testing services were often limited to clinical tests performed in the hospitals and health facilities with the National Reference Laboratory functionality basically restricted to performing limited testing services for epidemic-prone diseases such as measles, rubella and yellow fever. The lack of testing capacity in-country for Lassa fever, Dengue, Marburg and other haemorrhagic fevers affected the country’s health system during the onset of the EVD outbreak. This therefore added to the environment that enabled the rapid spread of EVD in a system with a weak diagnostics infrastructure and limited trained human resource capacity to combat such a devastating outbreak.

An extensive description of the pre-EVD laboratory structures and services, laboratory infrastructure and training institutions, including the laboratory-related gaps and key challenges in Liberia are further detailed in a corresponding publication entitled ‘Pre-Ebola virus disease (EVD) laboratory system and related challenges in Liberia’.^2^

## Post-Ebola virus disease laboratory strengthening initiatives

**FIGURE 1:**
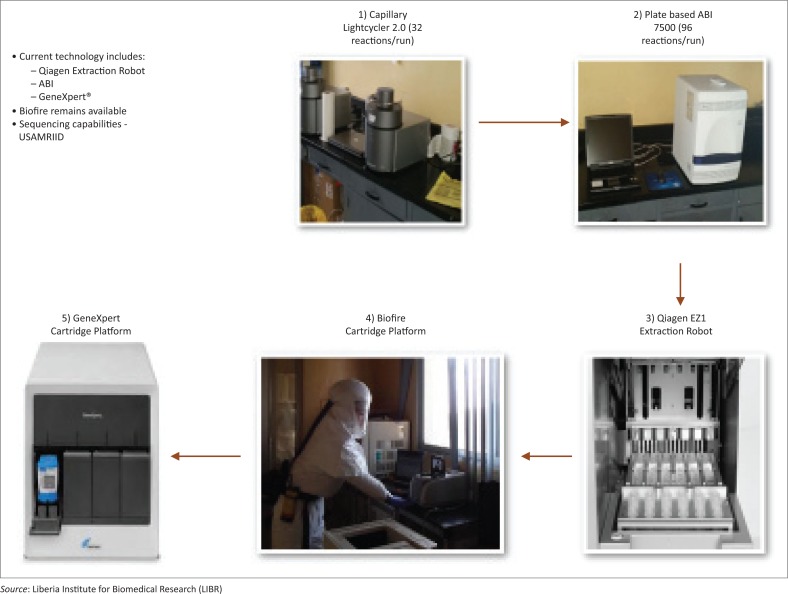
Post-Ebola virus disease technology advancement.

Between April 2015 and April 2016, there has been enhanced capacity to detect EVD and new approaches^3,4,5^ have been implemented (Figure 1). Several EVD-dedicated laboratories were set up across the country, in close proximity to an Ebola screening and/or Ebola treatment unit to inform admittance, treatment, and discharge decisions as well as to enhance routine surveillance of EVD.

**FIGURE 2:**
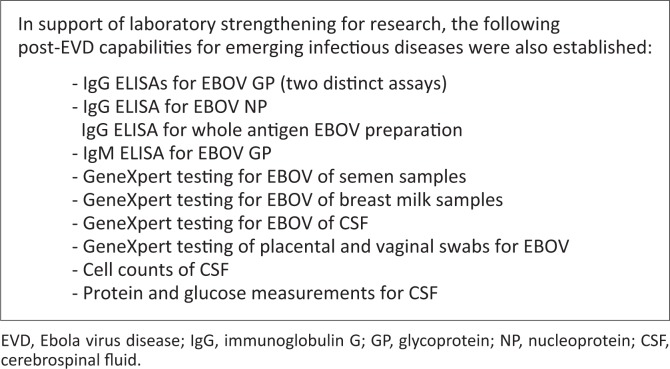
Research laboratory strengthening.

Biosafety and biosecurity considerations, as well as bio-risk management, were major strategic considerations employed during the determination for the establishment of an EVD testing laboratory in Liberia. The current EVD-dedicated laboratories in Liberia are biosafety level 2, with enhanced safety features for conversion to biosafety level 3 capabilities, including fluid-resistant positively-pressurised protective suits (Tyvek suits) with self-contained breathing apparatus, full face-shield or goggles, stringent decontamination procedures, and considerable oversight by laboratory managers, the Ministry of Health and the Incident Management System (IMS), the coordinating body for EVD response in the country. To date, these laboratories have shifted from the use of biosafety level 2 cabinets with a collapsible negative-pressure viral isolation chamber to a glove box, thereby reducing the level of personal protective equipment required and resulting in significant cost-savings. All the ‘hot suites’ in these laboratories have stringent work flow and security features (Figure 2).

The diagnostic options to enhance EVD surveillance had evolved since the outbreak in 2014. Traditionally, RNA detection using quantitative realtime PCR (qRT-PCR) within 3–10 days after the onset of symptoms had been used; however, the Liberian Ministry of Health recognised the need for a sensitive and selective rapid test to detect EVD at the point of care. Several point-of-care immunoassays (e.g., Corgenix’s ReEBOV™ Antigen Rapid Test; OraSure’s OraQuick) were granted the United States Food and Drug Administration’s emergency-use authorisation, and the World Health Organization listing for the presumptive detection of Ebola Zaire virus. These assays are able to detect Ebola virus (EBOV) antigen within 30 minutes. One of the regional laboratories in Liberia participated in the validation of the OraQuick^®^ rapid diagnostic test (RDT), using routine surveillance whole blood and oral fluid samples. Leveraging its simplicity and high containment, the Cepheid GeneXpert^®^ system is now the testing platform of choice by the Ministry of Health, having being validated elsewhere.^6^ This is an automated, single-cartridge system targeting the glycoprotein and nucleoprotein genes of EBOV, with test results available within 90 minutes. This system was selected over traditional qRT-PCR as being more user-friendly and sustainable in a good public–private partnership programme.

The use of the OraQuick Rapid Antigen Test was initiated in Liberia by the US Centers for Disease Control and Prevention, the World Health Organization and implementing partners in 2015, and was primarily used for surveillance of dead bodies during and after one of the sporadic clustered outbreaks in Liberia as the primary test, with qRT-PCR being the confirmatory test. The lessons learned in the implementation of the RDTs resulted in continued training on the use of the RDT by potential operators, such as laboratory technicians, nurses, clinicians, environmental health technicians, and mortuary staff. It was also determined that there was a need to periodically review the implementation process and devise innovative ways for verifying results, such as taking photographs of the RDT results for secondary independent analysis. The aim of this pilot was to build confidence in the use of the EBOV RDT in field situations as an EBOV infection screening test for dead bodies during an outbreak, then to extend its use to suspected cases and their contacts following further validation.

During the outbreak, capabilities for serology (breast milk, whole blood) and genomics were developed to help determine the source of an EVD cluster and EVD status.

At present, the 2016–2021 Laboratory Strategic Plan is being finalised. This strategy provides a road map for a robust and sustainable laboratory network throughout the country. An electronic Laboratory Information System is also under development to ensure that laboratory information is synthesised and utilised for timely decision making.

## Capacity-strengthening initiatives

In terms of workforce development, there has been significant investment in training and supervision, not only on the use of the equipment but also on preventive maintenance and servicing. Several Liberian laboratory technicians and technologists were initially trained on EBOV diagnostics by different agencies who were operating these laboratories. Now, there are at least 21 competent trained laboratory personnel in Ebola diagnostics. Each laboratory is staffed by five technicians, on average, and a data entry officer. The team is responsible for all aspects of infection, prevention and control, biosafety, biosecurity, sample reception, processing and testing, as well as interpretation and reporting of results. The laboratory staff have received extensive training on running a PCR laboratory and also in performing traditional qRT-PCR (whole blood, oral swab, vaginal swabs and semen) and troubleshooting.

**FIGURE 3:**
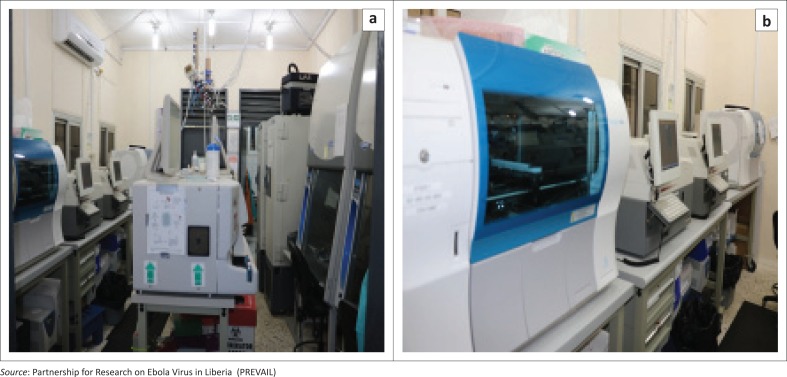
Post-Ebola virus disease technology enhancement at Redemption Laboratory.

Accordingly, there are now several EVD-dedicated diagnostic testing laboratories in Liberia: (1) the National Reference Laboratory in Margibi County; (2) the Tappita EVD Laboratory at Jackson F. Doe Hospital in Nimba County; (3) the ELWA Mobile Laboratory – GeneXpert only for whole blood samples; (4) Bong EVD laboratory at Phebe Hospital Complex in Bong County; and (5) the Redemption Hospital’s Central Laboratory that has been transitioned from international expert-directed to Liberian-led and expert-supported based on technical assistance and support, procurement of laboratory equipment and supplies, and inventory management (Figure 3). Approximately 18 technicians are currently undertaking training in biomedical equipment engineering such as servicing, maintenance and repair. A number of technicians have also been trained in GeneXpert system maintenance and calibration.

## Coordination of transport and referral services

An intricate and well-coordinated specimen referral (i.e., collection, transport and delivery) system supported by Riders for Health has also been established. The structure of the transportation and referral network conforms to universally-acceptable practices^7^ to ensure that specimens are delivered to the laboratory within a few hours to a maximum of 3–4 days post-collection. This network is also responsible for the distribution of specimens to laboratories within their daily testing capacities to ensure that samples are tested in a timely manner and the integrity is not compromised as a result of the transport and environmental conditions. This referral system not only supports EVD surveillance but also other diseases, as the Liberian health system shifts from the EVD emergency response to that of routine disease surveillance and preparedness. At present, specimen acceptance and rejection criteria are more stringent, based on sample eligibility criteria, unlike during the outbreak and the 90-day enhanced surveillance periods where efforts were directed at testing every specimen received by a laboratory, even if the patient identifiers were missing, as long as the presumed EVD status of the samples was determined.

## Lessons learned

Some of the laboratories in the country participated in the validation of oral swab, semen and vaginal fluid testing on the GeneXpert system in support of the EVD survivor programme. There have been lessons learned from each of the laboratories in terms of the gene targets and sensitivity of the various testing platforms when performing semen testing; therefore, care must be taken in the interpretation of the results when using traditional qRT-PCR instruments and approved Ebola assays. Given its sensitivity, the GeneXpert Ebola Assay has largely been used to resolve the observed discrepancies.

Redemption Hospital, a government-owned, free-service health facility, has adopted an integrated testing model since installation of the GeneXpert system in July 2015 for EBOV detection, with full implementation four months later. Cepheid’s Xpert Ebola Assay had been extensively validated for use with whole blood. This has benefited Redemption Hospital, which was among the hardest-hit hospitals, where triage and other important clinical decisions were made within two hours of receiving an EVD suspect’s specimen. Additionally, collaborations with other vertical programmes, such as the National AIDS Control Program and the National Tuberculosis & Leprosy Control Program, has been fostered, thereby translating to better use of limited resources such as shared equipment and laboratory staff. Plans are underway to roll-out the GeneXpert Systems in laboratories in some of the political sub-divisions of the country based on disease burden (e.g., HIV, tuberculosis); geographic location; patient density; and number of EBOV tests requested on a weekly basis.

### Conclusion and recommendations

The following recommendations are proffered to help mitigate challenges faced by the laboratory system:

1. Establishment of an integrated laboratory system with a well-coordinated mechanism for communication and interaction to ensure that duties and responsibilities are not duplicated. Such a system will likely reduce the confusion and safeguard needed resources in order to meet the national goals. Instead of multiple programmes, all laboratory or diagnostics functions should be coordinated under one umbrella.
2. Developing a comprehensive laboratory strategic plan and operational plan to meet current challenges is critical. The previous National Laboratory Strategic and Operational Plan was for the three years spanning 2011 to 2013. If done, this will guide the integrated laboratory system in aligning its stipulated goals with the country’s national health agenda and plans for rebuilding a resilient health system. Resources can then be sourced to support this comprehensive laboratory strategic plan.
3. Formulate a comprehensive training plan for upgrading the skills of laboratory personnel incorporated within the strategic plan. This should clearly indicate the training of laboratory personnel to become specialists in various areas ranging, for example, from immunology, microbiology, molecular biology, zoonosis, parasitology, biochemistry, biomedical engineering, virology, pathology, field epidemiology, laboratory management and disaster management, to laboratory information systems. In addition, capacity is needed at national universities and laboratory training institutions to provide advanced degrees (e.g., MSc, PhD) in laboratory programmes, support accreditation and continual education, and create a platform for career advancement in the field of laboratory medicine.
4. Develop EVD guidelines and standard operation procedures to regulate the donation of medical equipment, laboratory platforms and standardised laboratory equipment in-country, and solicit service contracts for the preventive maintenance and repair of broken equipment to ensure the continuity of diagnostic services. After standardisation, prioritise the issuance of contractual agreements to suppliers and manufacturers that have sales representatives and service centres in-country or within the sub-region, with restrictions stipulated regarding the time taken to respond to technical breakdown.
5. Ensure that biosafety and biosecurity, including quality control and assurance, become an integral part of the revised post-EVD national laboratory strategic and operational plan.

## References

[1] Ministry of Health and Social Welfare, Republic of Liberia National Diagnostics Unit: Strategic plan, 2011–2013. Monrovia, Liberia; 2011.

[2] Kennedy SB, Dogba JB, Wasunna CL, et al Pre-Ebola virus disease laboratory system and related challenges in Liberia. Afr J Lab Med. 2016;5(3), a508 http://dx.doi.org/10.4102/ajlm.v5i3.508 10.4102/ajlm.v5i3.508 PMC5433815 28879142

[3] Nouvellet P, Garske T, Mills HL, et al The role of rapid diagnostics in managing Ebola epidemics. Nature. 2015;528(7580):S109–116. http://dx.doi.org/10.1038/nature16041 26633764 10.1038/nature16041 PMC4823022

[4] Yen C-W, De Puig H, Tam JO, et al Multicolored silver nanoparticles for multiplexed disease diagnostics: distinguishing dengue, yellow fever, and Ebola viruses. Lab Chip. 2015;15(7):1638–1641. http://dx.doi.org/10.1039/c5lc00055f 25672590 10.1039/c5lc00055f PMC4375736

[5] Kaushik A, Tiwari S, Dev Jayant R, et al Towards detection and diagnosis of Ebola virus disease at point-of-care. Biosens Bioelectron. 2015;75:254–272. http://dx.doi.org/10.1016/j.bios.2015.08.040 26319169 10.1016/j.bios.2015.08.040 PMC4601610

[6] Schnippel K, Meyer-Rath G, Long L, et al Scaling up Xpert MTB/RIF technology: the costs of laboratory- vs. clinic-based roll-out in South Africa. Trop Med Int Health. 2012;17(9):1142–1151. http://dx.doi.org/10.1111/j.1365-3156.2012.03028.x 22686606 10.1111/j.1365-3156.2012.03028.x PMC3506730

[7] US Centers for Disease Control and Prevention Guidance for collection, transport and submission of specimens for Ebola virus testing. Updated January 2015. Atlanta, GA: CDC; 2015.

